# Ultrastructure and fractal property of chromosomes in close-to-native yeast nuclei visualized using X-ray laser diffraction

**DOI:** 10.1038/s41598-023-37733-6

**Published:** 2023-07-05

**Authors:** So Uezu, Takahiro Yamamoto, Mao Oide, Yuki Takayama, Koji Okajima, Amane Kobayashi, Masaki Yamamoto, Masayoshi Nakasako

**Affiliations:** 1 Department of Physics, Faculty of Science and Technology, Keio University, 3-14-1 Hiyoshi, Kohoku-Ku, Yokohama, Kanagawa 223-8522 Japan; 2 RIKEN, Spring-8 Center, 1-1-1 Kouto, Sayo-Cho, Sayogun, Hyogo 679-5148 Japan; 3 PRESTO, Japan Science and Technology Agency, Chiyoda-Ku, Tokyo, 102-0076 Japan; 4 Graduate School of Science, University of Hyogo, 3-2-1 Kouto, Kamigori-Cho, Ako-Gun, Hyogo, 678-1297 Japan; 5 International Center for Synchrotron Radiation Innovation Smart, Tohoku University, Katahira 2-1-1, Aoba-Ku, Sendai, 980-8577 Japan; 6 CRESTO, Japan Science and Technology Agency, Chiyoda-Ku, Tokyo, 102-0076 Japan

**Keywords:** Biophysics, Structural biology

## Abstract

Genome compaction and activity in the nucleus depend on spatiotemporal changes in the organization of chromatins in chromosomes. However, the direct imaging of the chromosome structures in the nuclei has been difficult and challenging. Herein, we directly visualized the structure of chromosomes in frozen-hydrated nuclei of budding yeast in the interphase using X-ray laser diffraction. The reconstructed projection electron density maps revealed inhomogeneous distributions of chromosomes, such as a 300 nm assembly and fibrous substructures in the elliptic-circular shaped nuclei of approximately 800 nm. In addition, from the diffraction patterns, we confirmed the absence of regular arrangements of chromosomes and chromatins with 400–20 nm spacing, and demonstrated that chromosomes were composed of self-similarly assembled substructural domains with an average radius of gyration of 58 nm and smooth surfaces. Based on these analyses, we constructed putative models to discuss the organization of 16 chromosomes, carrying DNA of 4.1 mm in 800 nm ellipsoid of the nucleus at the interphase. We anticipate the structural parameters on the fractal property of chromosomes and the experimental images to be a starting point for constructing more sophisticated 3D structural models of the nucleus.

## Introduction

The genetic information recorded in the genomic DNA is implemented in cellular activities through spatiotemporal changes in the organization of a large number of chromatins, assemblies of DNA and proteins, in chromosomes, and the patterns of gene positioning affect the transcription status of the genes^1–3^. In the nuclei of eukaryotic cells, individual gene loci are believed to occupy preferential positions with respect to their chromosome territory (CT) and/or other nuclear landmarks such as the nuclear envelopes and nucleoli^4^. Although the spiral distribution of DNAs stored in a bacteriophage capsid was visualized by transmission electron microscopy (TEM)^5^, the compaction mechanism of chromatins in the eukaryotic nuclei is probably more complicated than that observed in virus particles. Although computational models proposed regarding the spatial organization of chromosomes in the nucleus after genome sequencing^6^, imaging studies are necessary to elucidate spatiotemporal variations in the organization and three-dimensional (3D) structures of chromosomes and chromatins in the nuclei^7^.

Various imaging techniques have been applied to understand the structures and dynamics of building blocks in chromatin assemblies at the molecular level. TEM observations of isolated nucleosomes have revealed a variety of higher-order structures depending on the solution conditions, and have led to different ideas on models of nucleosome assemblies^7,8^. A TEM study of chemically labeled human chromosomes reported that flexible and structurally heterogeneous chains of 5–24 nm diameter are packed together without any higher-order structures^9^. Imaging studies using super-resolution light microscopy proposed the presence of nucleosome clutches, which are heterogeneous groups of nucleosomes leading to the formation of chromatin in human nuclei^10^, a power law between the size and length of chromosomes in Drosophila nuclei^11^, and compact domains with a diameter of approximately 160 nm in vertebrate nuclei^12^. In addition, fluorescence in situ hybridization was used to illustrate the spatial organization of more than two loci^13^.

An alternative approach to visualize the organization of chromosomes is the high-throughput chromosome conformation capture (Hi-C) technique^14,15^, which provides structural information on the arrangement of chromosomes, such as long-range interactions of chromatins, at each genomic locus^16^ and at the resolution of nucleosome level^17–19^. Studies using the Hi-C techniques have revealed the characteristic features on higher-order folding of chromatins in chromosomes, such as a broad segregation of the genome into compartments^20^, and topologically associated domains (TADs) of hundreds of kilobases^21–23^. Based on the distance geometry information of the loci, the 3D distributions of chromosomes were computationally derived^24,25^.

Three-dimensional structures of whole cells have been studied using TEM for thin sections^26^, high-voltage cryoTEM tomography^27^, and focused-ion beam scanning electron microscopy^28^. In TEM observation, as the contrast of DNA was poor against the other components in nuclei, chromatins were difficult to be identified unambiguously in frozen-hydrated nuclei as pointed out^9^.

The techniques described above are useful for studying the structures and dynamics of chromosomes, but require physicochemical modification and/or treatment of the nuclei. Super-resolution microscopy has achieved spatial resolution higher than 100 nm^29^, but fluorescent labels are necessary to visualize specific genomic loci. In addition, only the positions of the labeled molecules are visualized. Regarding the Hi-C technique^24^, the influence of formamide-crosslinking on the native chromatin organization is still unclear. In electron microscopy observation except high-voltage cryoTEM, specimens are stained by osmium reagent in the presence of acetone^28^ or sliced after the fixation using epoxy resin^26^.

In contrast, X-ray imaging is a potential technique to visualize the structure of the whole nuclei without sectioning and chemical labeling. Soft X-ray imaging was applied to the structural study of frozen-hydrated eukaryotic cells^30–33^. The imaging technique is advantageous to visualize the differences in X-ray absorption of substances of cells. However, as the absorption contrast is poorer than the electron density contrast measurable in X-ray diffraction using short-wavelength X-ray, the visualization of the structural organization of chromosomes in the nuclei regarding X-ray absorption contrast may be still under progress.

X-ray diffraction imaging (XDI)^34^, a lens-less imaging technique, helps in the visualization of the shape, size, and internal structure of non-crystalline particles as electron density without the need of sectioning and chemical labeling, owing to the penetration power of X-rays with short wavelengths. XDI has been applied to investigate the structures of frozen-hydrated biological cells with sizes ranging from sub-micrometer to several micrometers using synchrotron X-rays^34,35^. The recent implementation of X-ray free electron laser (XFEL), which provides very intense and spatially coherent X-ray pulses at high repetition rates, enables the rapid collection of diffraction patterns from a large number of non-crystalline specimens, such as metal particles^36,37^, organelles^38^, and biological cells^39^, the sizes of which are smaller than the cross-section of the XFEL pulses. As the electron density contrast of a specimen are reconstructed from the diffraction patterns, XDI structure analysis of the nuclei may be advantageous to visualize the distribution of chromosomes with the highest electron density contrast among the substances in the nuclei.

**Figure 1:**
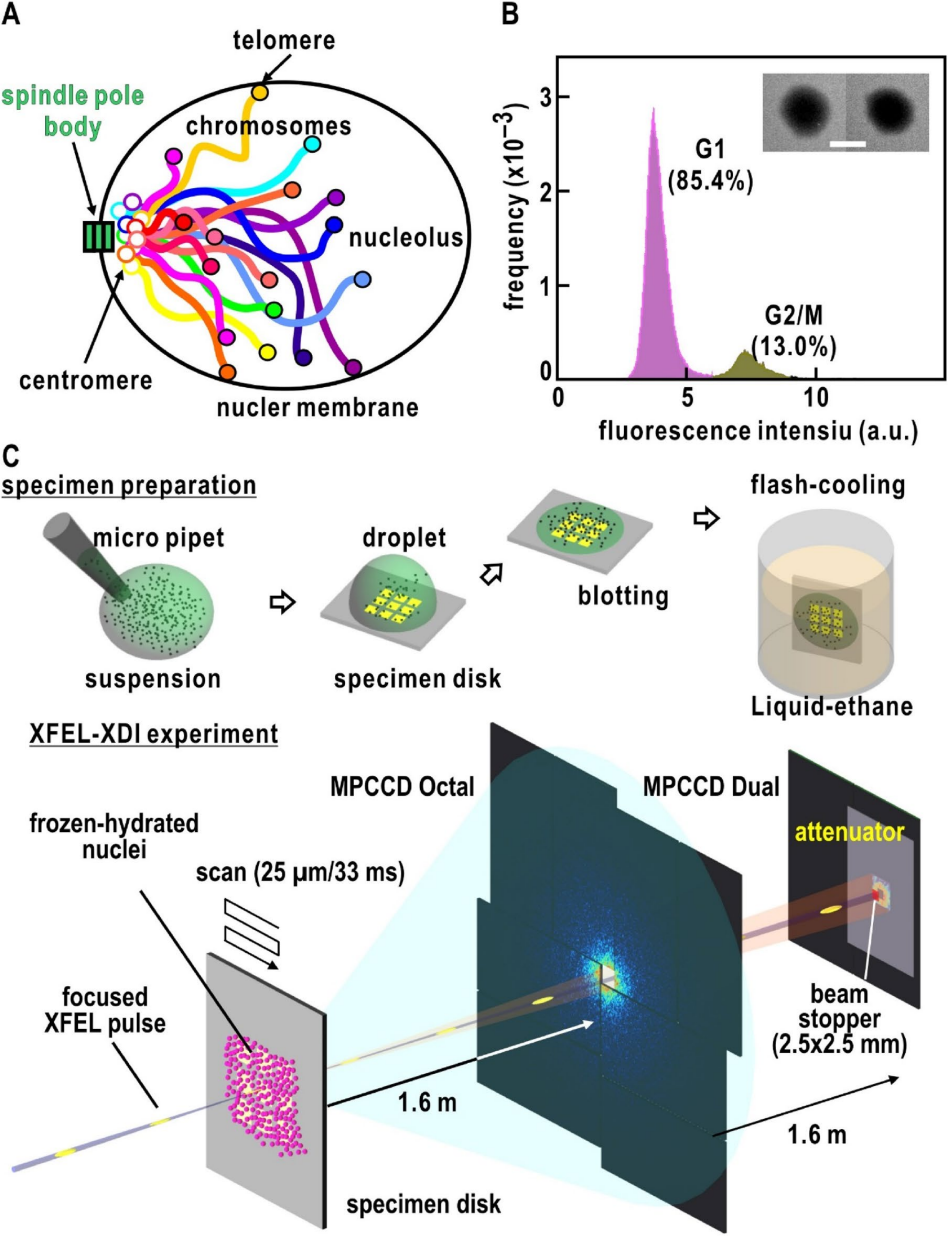
(**A**) Hypothetical illustration on the distribution of 16 chromosomes (I-XVI) in the Rabl configuration in the yeast nuclei in the interphase ^42–44^. (**B**) Flow cytometry analysis for yeast cells cultured in the presence of raffinose. Inset is TEM images of the nuclei with the scale bar of 400 nm. (**C**) Schematic illustrations on the specimen preparation and XFEL-XDI experiments at SACLA ^34,36–39^.

In this study, we investigated the close-to-native structure of chromosomes in frozen-hydrated nuclei of the budding yeast *Saccharomyces cerevisiae* at the interphase^40^ using XFEL-XDI. As the structures of chromosomes in nuclei have been investigated for various biological species of eukaryote, it is interesting whether any structural architectures in *S. cerevisiae* are common among the species. The budding yeast *S. cerevisiae* is an attractive model system to study nuclear organization and its functional relevance^41^. At the interphase, 16 chromosomes with different DNA composition^42^ (SI Appendix, Table S1) are organized in a Rabl-like chromosome configuration^43,44^, where the centromere of each chromosome is tethered by the spindle pole body (SPB)^45^, a single microtubule and the kinetochore complex to a multiprotein complex embedded in the nuclear envelope, and the telomeres are tethered to the nuclear envelope (Fig. 1A). The nucleolus, a platform for the transcription of ribosomal RNAs and the construction of ribosomes, occupies the outside of the CT near the opposite side of the SPB.

The diffraction patterns and reconstructed electron density maps indicated that the chromosomes were composed of self-similarly arranged substructural domains in non-uniform distribution. Based on the structural information, we discuss how 16 chromosomes carrying DNA with a total length of approximately 4.1 mm are organized in the nuclei of an approximately 800 nm diameter.

## Results

### XFEL-XDI

Nuclei were isolated from raffinose-arrested yeast culture, in which more than 85% of cells were in the interphase as measured by flow cytometry analysis (Fig. 1B and the “Methods” section). From a TEM observation, the size of the nuclei was approximately 800–1000 nm and the shapes were approximated as ellipsoid (Fig. 1B). Isolated nuclei were randomly dispersed on Si_3_N_4_-membrane windows of a specimen disk and flash-cooled using liquid ethane (Fig. 1C)^46^.

In XFEL-XDI experiments, diffraction patterns of the nuclei were collected by supplying frozen-hydrated nuclei into the irradiation area of the focused XFEL pulses by scanning the specimen disk (Fig. 1C and the “Methods” section)^47^. Diffraction patterns were recorded by tandemly placed two multiport charge-coupled device (MPCCD) detectors and automatically processed using a custom-made data processing software suite^48^. Although a focused XFEL pulse destroys a specimen particle at the atomic level, diffraction occurs from the particle before its destruction^49^.

**Figure 2:**
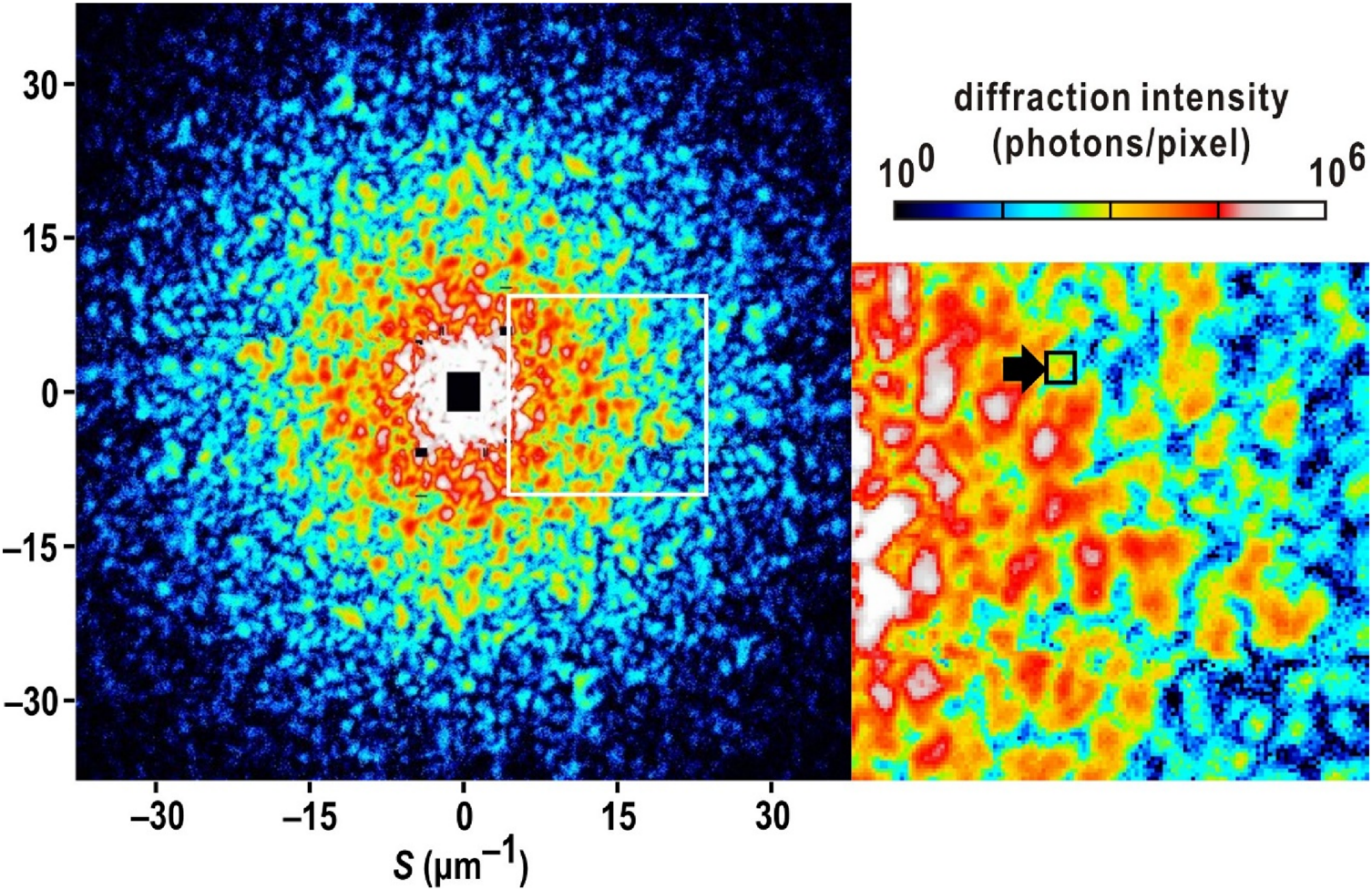
Typical diffraction pattern from the yeast nucleus in the interphase. The magnitude of the scattering vector is defined as $S = {{2\sin \theta } \mathord{\left/ {\vphantom {{2\sin \theta } \lambda }} \right. \kern-0pt} \lambda }$, where $2\theta$ is the diffraction angle and $\lambda$ is the X-ray wavelength. The right panel is a magnified view of the diffraction pattern (indicated by a white box in the left panel) to demonstrate the good visibility of each speckle peak. The black box with an arrow indicates a small speckle corresponding to the reciprocal of the size of a nucleus.

In two independent XFEL-XDI experiments, we extracted 1,333 diffraction patterns satisfying the following two criteria for subsequent analyses: signal-to-noise ratios higher than 3 at a resolution of 33 nm (corresponding to a resolution of 30 μm^‒1^ in diffraction space), good visibility of the interference peaks (Fig. 2), and high centrosymmetry (SI Appendix Fig. S2A) as described in the “Methods” section. Figure 2 shows a representative diffraction pattern from a single nucleus of the interphase (see also SI Appendix, Fig. S1). The widths of the narrow interference peaks in diffraction patterns were comparable with the reciprocal of the nuclei sizes observed in the TEM image (Fig. 1B) and those observed in yeast cell by soft X-ray imaging^30^. Although nuclei in the Gap2/Mitosis (G2/M) phase^41^, which had larger sizes and amounts of DNAs than those of the interphase, were contained in the specimen suspension (Fig. 1B), the diffraction patterns of those nuclei were excluded by the strong diffraction intensity beyond the dynamic range of the detectors. Larger sizes of the nuclei made their diffraction patterns finer than those from the nuclei in the interphase. Finer patterns result in smaller oversampling ratios to hinder the reconstruction of electron density map.

### Shapes and sizes of the nuclei

**Figure 3:**
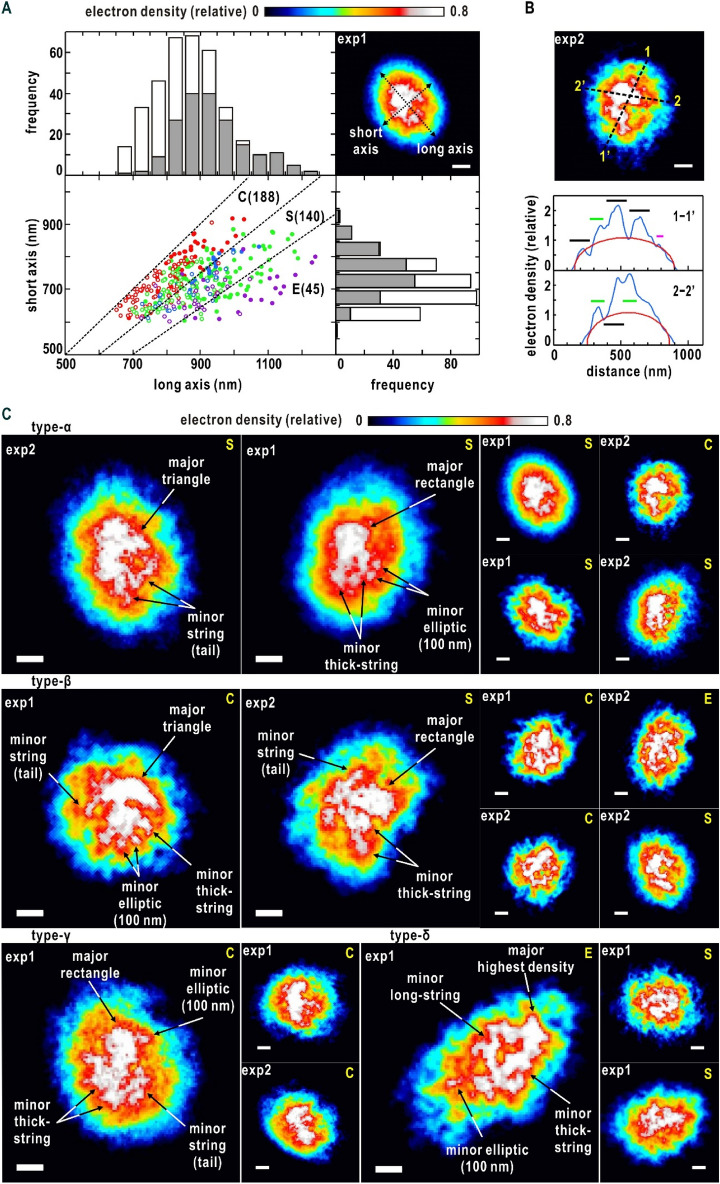
(**A**) Size distribution of the projection maps approximated as elliptic circles illustrated in the top right panel. Open and filled circles indicate the size of the maps from the first (exp1) and second experiments (exp2). In addition, the symbols are colored with respect to the internal fine structures type-α (red), type-β (green), type-γ (blue) and type-δ (purple) shown in panel (**C**). In the frequency distributions of the long and short axes, the gray and white bars indicate the data from the first and second experiment, respectively. The dashed lines define the borders regarding the ARs of elliptic circles approximating the map shapes as circular shapes ‘C’ (1 < AR < 1.2), semi-circular shapes ‘S’ (1.2 < AR < 1.4), and elliptic shapes ‘E’ (1.4 < AR). The number of maps composing each category is shown in parenthesis. Here after, the labels at the upper left corner of each map indicates the first/second experiment (exp1/expp2), and the scale bar in each map indicates 200 nm. (**B**) Electron density profiles in a representative map (upper panel). The blue curves show the profiles along the dashed lines. The red curves are the profiles of projection density of ellipsoids with uniform density^52^. Peaks with approximate width of 150, 100, and 50 nm are indicated by black, green, and magenta bars, respectively. (**C**) Representative projection electron density of the types-α, β, γ, and δ maps. In each map, the label at the top right corner indicates the shape of the map classified according to the AR in panel (**A**). Characteristic fine-structures of major and minor high-density regions described in the main text are labeled in the magnified views of representative projection maps.

We reconstructed 1333 maps from extracted diffraction patterns at a resolution of 25 nm (corresponding to a resolution of 40 μm^‒1^ at the edge of diffraction pattern), the highest resolution achievable by our experimental setup (see Fig. 1C and the “Methods” section). At the resolution, the curvature of the Ewald sphere has little influence on the projection map as reported previously^50^. Among the reconstructed maps, 373 maps were extracted as projection views of electron density distribution in the nuclei through examining the similarity scores of a set of retrieved maps (SI Appendix Fig. S2B,C)^39,51^, the consistency of overall shapes of nuclei in the maps with the TEM observation in Fig. 1B and soft X-ray imaging for the nucleus inside a yeast cell^30^ (Fig. 3A), and the continuous variation of density inside nuclei (Fig. 3B,C) (see the “Methods” section). The blurred boundary of the projection map was predominantly attributed to the electron density contrast of the nuclei against vitreous ice^52^ (SI Appendix, Fig. S3).

The whole shapes of the nuclei in the retrieved maps were approximated as ellipses (Fig. 3). When classifying the maps with respect to the aspect ratios (ARs) between the long and short axes of ellipses approximating the whole shapes (Fig. 3A), circular (1.0 < AR < 1.2) and semicircular shapes (1.2 < AR < 1.4) were the major contributors. Regarding the circular and semicircular shapes, the size distributions of maps almost overlapped between the two independent experiments (Fig. 3A). In addition, the shapes and sizes were consistent with those in TEM images (Fig. 1B) and in the 3D image of a yeast cell visualized by soft X-ray imaging^30^. Therefore, the nuclei with the AR < 1.4 were deemed free of any fatal damages such as puncture and/or heavy deformation during the preparation. In contrast, elliptic shapes (1.4 < AR) formed a minor fraction and predominantly appeared in the first experiment only and were slightly inconsistent with the images observed in the other imaging techniques. The elliptic-shaped nuclei may be deformed in blotting procedure in the specimen preparation (Fig. 1C) rather than in the inclination of the nuclei against the incident X-ray.

The variety in the shapes and sizes in AR < 1.4 was mainly attributed to the orientation of nuclei on the specimen disks against the direction of the XFEL pulses. The frequency distribution suggested that the 3D shapes of the nuclei in the interphase in AR < 1.4 could be approximated as a prolate spheroid, and the average half-lengths of the long and short axes were 422 nm and 340 nm, respectively.

### Fine structures in nuclei

Projection maps displayed inhomogeneous density distribution (Fig. 3B); the density profile was composed of a smoothly varying part expected from the projection of an ellipsoid with a uniform density^52^ and a fluctuating part exceeding the smooth part. Chromosomes/chromatin have the highest electron density among the substances composing the nuclei, and RNA-rich regions may contribute to high electron-density area depending on the density. When taking the shape and size of the high electron density regions described below, chromosomes and their assemblies were probably the predominant components of the high-density areas. When inspecting the pronounced electron density fluctuation, peaks with widths ranging 50–150 nm suggested the presence of local fine structures, where chromatin/nucleosomes were condensed (Fig. 3B).

Here, we focus on the distribution patterns of high-density regions. In each projection map, a single high-density region with triangular or rectangular shapes of approximately 300 × 300 nm occupied the region 100–200 nm apart from the center of the whole nucleus (Fig. 3C). In contrast, high densities were rarely observed near the nuclear envelops. With respect to the locations and shapes of the major and minor high-density regions, maps were roughly classified into four types, namely α, β, γ, and δ, by visual inspection (Fig. 3). The number of maps in the α, β, γ, and δ were 123, 163, 39 and 48, respectively. In addition, the shapes, sizes, and relative locations of the major/minor densities of the maps were similar between the two independent experiments, suggesting the presence of any specific organization patterns of chromosomes in the nuclei at the interphase.

Type-α maps appeared in 112 circular and 11 semicircular shapes (Fig. 3A and C). Among the four types of maps, type-α maps displayed characteristic distributions of high-density regions (Fig. 3C). Regarding the locations of the major and minor density peaks, type-α maps were divided into two representative patterns. In the most characteristic pattern, a few string-shaped (or fibrous) densities of approximately 50 nm width, such as tails, protruded from the major density. In the other, a few elliptic density peaks with approximately 100 nm diameter were distributed around the major density. Compactly assembled chromosomes around the SPB in the Rabl configuration may be responsible for the major density, and five chromosomes IV VII, XII, XV and XVI (SI Appendix, Table S1), each carrying approximately one million DNA base pairs, may assume string-shaped structures.

Type-β maps, which appeared in 62 circular, 79 semicircular and 22 elliptic shapes (Fig. 3A and C), were most frequently observed among the 373 maps. Minor densities of 200–300 nm long and 100 nm wide were distributed in contact with the edges or sides of the major densities (Fig. 3C), and frequently seemed to radiate from the major densities. According to the Rabl configuration in the interphase, the centromeres of 16 chromosomes were distributed around the SPB, which has a multi-layered structure with a shape to cylindrically arrange microtubules^45^. When viewing the nuclei from the SPB side, the projection densities around SPB may yield the major density regions and the chromosomes extended to the telomeres may appear as the minor densities as in the type-β maps.

Type-γ maps appeared in 12 circular, 26 semicircular shapes and additionally in one elliptic shape (Fig. 3A and C). In contrast to type-α and type-β maps having single major density regions, type-γ maps were characterized by a few major density regions separated by approximately 200–350 nm from each other. Minor densities filling the gaps between the major densities and string-shaped densities protruded from the major densities.

As type-δ maps were found in 3 circular, 24 semicircular and 21 elliptic shapes and were predominantly observed in the first experiment (Fig. 3A and C). In the type-δ maps, a few string-shaped densities of 300–500 nm long were extended from the highest density peak in a parallel or crossed arrangement. The long string-shaped densities comprised density peaks with an approximate diameter of 50 nm. The thick string-shaped densities may correspond to bundles of long chromosomes, such as IV, VII an XII (SI Appendix. Table S1). As approximately half of the type-δ maps had elliptic shapes (AR > 1.4), which might be deformed in the specimen preparation (Fig. 3A), the type-δ maps were excluded in the following analysis.

### Fractal property in chromosome organization

**Figure 4:**
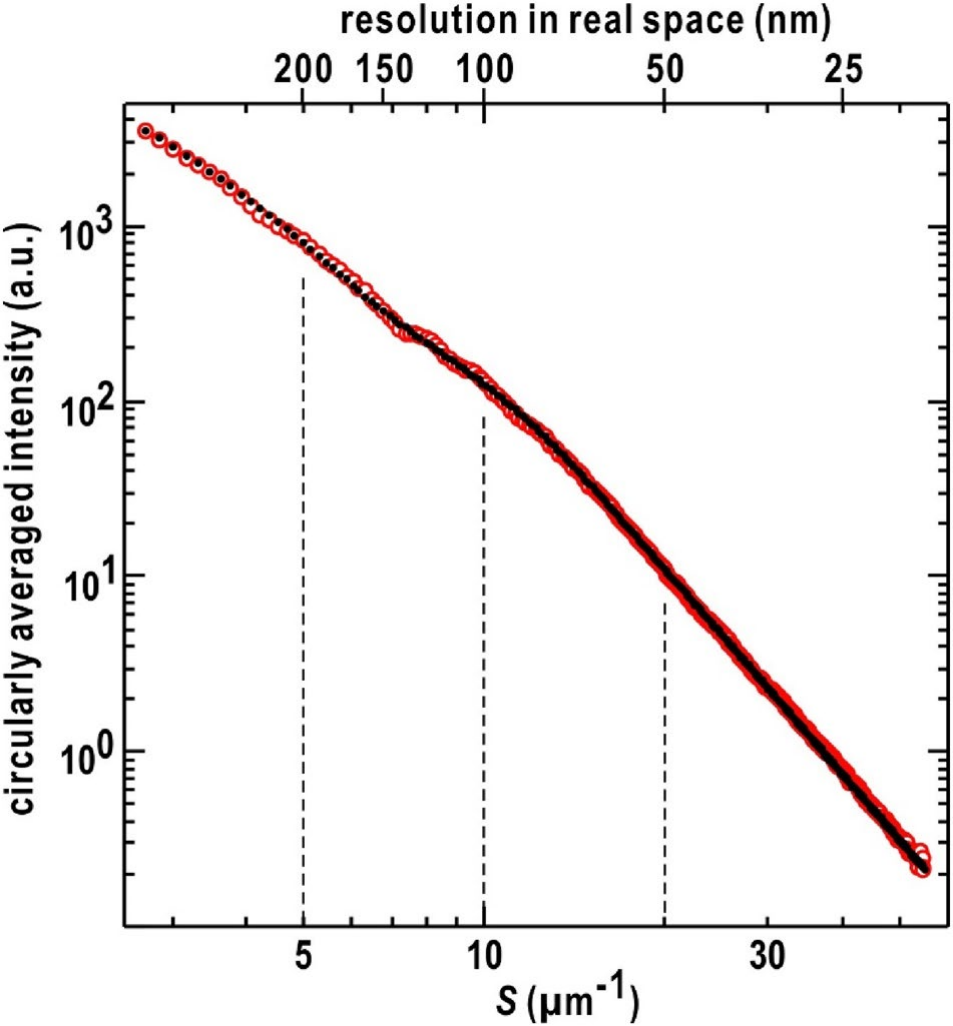
Diffraction profile obtained by summing the circularly averaged diffraction patterns (red open-circles) and the best fit of the general scattering function of Eq. (1)^53^ (black dots). As the generalized scattering function is complicated and non-linear, we independently performed ten fitting calculations, and the deviation of each fitting curve from the experimental profile was evaluated by the residual factor defined as $R_{i} = {{\sum\limits_{{S \in {\text{region}}i}} {\left| {I_{{{\text{obs}}}} \left( S \right) - I_{{{\text{fit}}}} \left( S \right)} \right|} } \mathord{\left/ {\vphantom {{\sum\limits_{{S \in {\text{region}}i}} {\left| {I_{{{\text{obs}}}} \left( S \right) - I_{{{\text{fit}}}} \left( S \right)} \right|} } {\sum\limits_{{S \in {\text{region}}i}} {I_{{{\text{obs}}}} \left( S \right)} }}} \right. \kern-0pt} {\sum\limits_{{S \in {\text{region}}i}} {I_{{{\text{obs}}}} \left( S \right)} }}$, where $I_{{{\text{obs}}}} \left( S \right)$ and $I_{{{\text{fit}}}} \left( S \right)$ are the experimental and theoretical profiles, respectively. The residual factor was calculated for four independent regions with different intensity ranges (I: *S* < 5, II: 5 < *S* < 10, III: 10 < *S* < 20, and IV: 20 < *S* < 55 μm^−1^) as indicated by the dashed lines. In the best-fit case, the factor values were 0.025, 0.037, 0.021, and 0.024 for regions I– IV, respectively.

The electron density fluctuation in the profiles of the projection maps suggested that certain substructures existed in the nuclei at the interphase (Fig. 3C). To address substructures common among the nuclei, we created a diffraction profile (Fig. 4) by summing circularly averaged diffraction patterns, which displayed good signal-to-noise ratios up to a resolution of 50 nm (corresponding to a resolution of 20 μm^‒1^ in diffraction space) and yielded maps of the ARs smaller than 1.4. As the selected 43 diffraction patterns yielded 17 type-α, 19 type-β and 7 type-γ maps, the shapes of which were circular or semicircular, the averaged profiles excluded structure information from elliptic shaped nuclei, which may be in slight deformation.

The profile displayed no diffraction maxima in the resolution range of 400–20 nm (corresponding to a resolution range of 2.5–50 μm^−1^ in diffraction space), indicating that any major and specific arrangements of chromosomes and/or chromatins were poor in the spacing of 400–20 nm. Since each diffraction pattern came from a single nucleus, the averaged profile was substantially different from the diffraction pattern of a pellet of nuclei, in which inter-nuclei interference of X-rays significantly modifies the diffraction patterns from each nucleus.

The profile was approximated by two regression lines on the border of an inflection located at approximately 10 μm^−1^ (Fig. 4); this was similar to those from self-similarly assembled synthetic polymers, which were analyzed by the generalized scattering function^53^. The function at the scattering vector length *S* is

1 $$\begin{gathered} I\left( S \right) \propto A\exp \left( { - \frac{{4\pi^{2} }}{3}S^{2} Rs^{2} } \right) \times \left\{ {{{\left[ {erf\left( {\frac{2\pi S}{{\sqrt 6 }}R{\text{g}}} \right)} \right]^{3} } \mathord{\left/ {\vphantom {{\left[ {erf\left( {\frac{2\pi S}{{\sqrt 6 }}R{\text{g}}} \right)} \right]^{3} } {2\pi S}}} \right. \kern-0pt} {2\pi S}}} \right\}^{{D_{{\text{m}}} }} \hfill \\ \, + B\exp \left( { - \frac{{4\pi^{2} }}{3}S^{2} R{\text{s}}^{2} } \right) + C\left\{ {{{\left[ {erf\left( {\frac{2\pi S}{{\sqrt 6 }}R{\text{s}}} \right)} \right]^{3} } \mathord{\left/ {\vphantom {{\left[ {erf\left( {\frac{2\pi S}{{\sqrt 6 }}R{\text{s}}} \right)} \right]^{3} } {2\pi S}}} \right. \kern-0pt} {2\pi S}}} \right\}^{{\left( {6 - D_{{\text{s}}} } \right)}} \hfill \\ \, erf\left( a \right) = \frac{2}{\sqrt \pi }\int\limits_{0}^{a} {\exp \left( { - t^{2} } \right){\text{d}}t} , \hfill \\ \end{gathered}$$

where *R*g and *R*s are the radii of gyration of the whole particle and substructural domains, respectively. *D*_m_ is the mass fractal dimension regarding the packing mode of the self-similarly arranged substructural domains, and *D*_s_ is the surface fractal dimension reflecting the surface roughness of the domain. *A, B*, and* C* are the constants.

For the nuclei, we assumed that the whole shape of the nucleus could be approximated by a prolate ellipsoid with the long axis being 422 nm and the short axis being 340 nm (Fig. 3A), yielding a radius of gyration, *R*g, of 286 nm using the following relation^54^:

2 $$R{\text{g}} = \sqrt {\frac{{2a^{2} + b}}{5}} ,$$

where *a* and *b* are the short and long axes of the prolate ellipsoid. It should be noted that the fitting parameters *R*s, *D*_m_ and *D*_s_ were robust for given *R*g values larger than 250 nm, because the error function in the first term of Eq. (1) is almost 1 in the region for fitting.

The experimental profile was reproduced by Eq. (1), when the substructural domains, having an average *R*s of 58 ± 2 nm and small surface roughness resulting in *D*_s_ of 2.1 ± 0.1, were self-similarly organized to yield *D*_m_ of 2.1 ± 0.2 (Fig. 4), with small differences between the experimental and theoretically predicted profiles (see the caption of Fig. 4). If the shape of a domain is a sphere, the diameter ($\sqrt {{5 \mathord{\left/ {\vphantom {5 3}} \right. \kern-0pt} 3}} Rs$) is 150 ± 5 nm. This size was comparable with the observed period of electron density profiles (Fig. 3B). The profile and the parameters provided the restraints for arranging the self-similarly organized substructural components in chromosomes.

## Discussion

**Figure 5:**
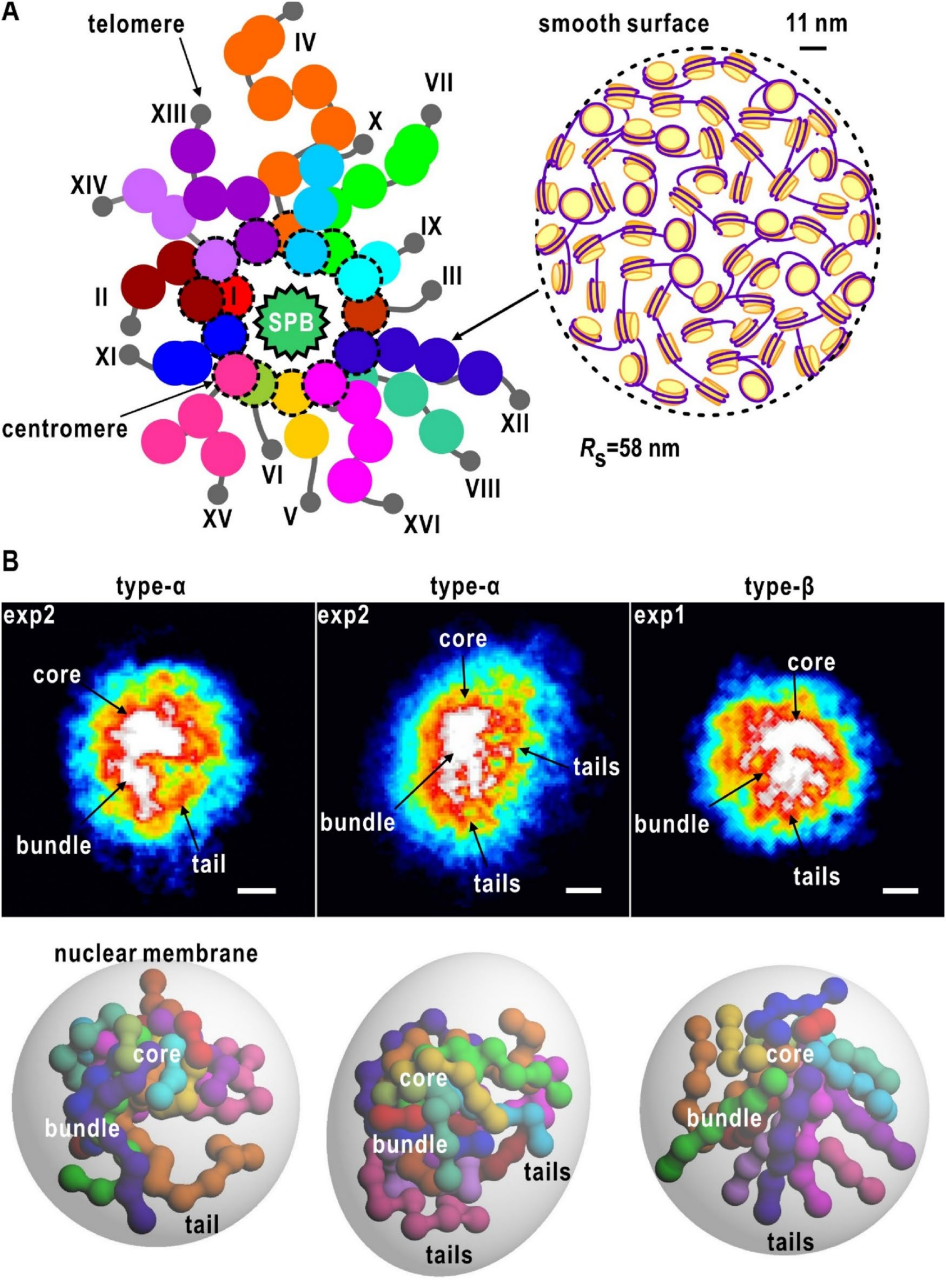
(**A**) Schematic illustration depicting the self-similar organization, like a Lichitenberg figure, of 16 chromosomes composed of substructural domains containing a number of nucleosomes. (**B**) Putative models on the organization of chromosomes to explain the representative projection electron density maps. The chromosome models are colored differently as in panel (**A**).

In the present study, we visualized the projection electron density maps of the *S. cerevisiae* nuclei in the interphase (Fig. 3). The overall shapes of the projection maps were approximated as elliptic circles, suggesting the ellipsoidal shapes of the nuclei. From the fine structures in the electron density distributions, the presence of substructural domains in chromosomes were suggested and further analyzed for the profile averaged over high-quality diffraction patterns. The analysis indicated no regular structures in chromosomes but the fractal nature in the structure and arrangements of the substructural domains (Fig. 4). Based on the structure analysis, we discuss the structural organization of chromosomes in the nucleus in the interphase (Fig. 5).

The models to speculate the structural organization of chromosomes were constructed to explain both the electron density distribution in the projection maps (Fig. 3) and the structural parameters obtained from the diffraction profile (Fig. 4). The major components of the substructural domains are the nucleosomes, which has 11 nm diameter and 6 nm thickness, and carries approximately 150 DNA base pairs^7^. If the substructural domain is a sphere of 150 nm diameter, that is suggested from the *R*s of the single substructural domain, the volume of the domain is capable of storing approximately 3000 nucleosomes carrying 0.45 mega bases of DNA in the closest-packing mode (Fig. 5A). Using this model of the single substructural domain, each chromosome was simply modeled as an assembly of spheres with 150 nm diameter. Then, the length of a chromosome depended on an amount of contained DNA. Nucleosomes at the periphery of the substructural domains may be arranged to form smooth surfaces based on the experimentally determined *D*_s_ value (Fig. 4). In addition, various types of higher-order structures of nucleosomes^7,8^ may exist inside domains; however, none of the higher-order structures are regularly arranged and also dominant to yield the observed diffraction profile without diffraction maxima in the spacing of 400–20 nm (Fig. 4).

The observed *D*_m_ value would be possible roughly in two types of chromosomal arrangements. One is the distribution of substructural domains like a randomly branched tree and Lichenberg figure as observed in synthetic polymers^55^, and the other is the condensation of domains in the vicinity of the nuclear envelope. The distributions of high-density regions in the middle of the projection maps (Fig. 3) ruled out the possibility of the latter. Therefore, as schematically illustrated in Fig. 5A, we envisage that the domains are arranged to mimic a randomly branched tree and also to avoid diffraction maxima in the resolution range of 400–150 nm. Then, the fractal dimensions would be helpful as new restraints, for instance, in model construction for the structural organization of chromosome and chromatin distributions^6,56^.

Based on the structural information described above and the Rabl configuration in the interphase of *S. cerevisiae*, we speculated the structural organization of the chromosomes in the nucleus in the interphase to explain three representative projection maps of types-α and β (Fig. 5B). Each chromosome was modeled as tandemly arranged spherical domains of 150-nm diameter, which represented the substructural domain with the *R*s value of 58 nm. The number of the spherical domains was proportional to the length of DNA contained in each chromosome, and the spherical domains were assumed to flexibly change the mutual positions. Then, to satisfy the Rabl configuration, one of the edges of each chromosome model was set near an assumed SPB, and the other was set near the nuclear envelope, which was approximated as a prolate spheroid with the half-lengths of the long and short axes of 422 nm and 340 nm, respectively. As chromosomes were arranged to explain the projection maps, each model was composed of a core region, bundles, and tails (Fig. 5B), and the three regions may be observed as CT. The core region was an assembly of the centromere regions of the 16 chromosomes gathered around the SPB and explained the highest density in the projection maps. Large chromosomes, such as IV, VII, XII, XIII, XV, and XVI, containing long DNA may be responsible for the bundles and tails radiating from the core region. In addition, the calculated *D*_m_ values for the arranged substructural domains in the putative models were approximately 2. In contrast to the type-α and type-β maps (Fig. 5B), at the present time, the interpretation of type-γ maps (Fig. 3C) was difficult because of the presence of a few high-density regions.

As our cryogenic XFEL-XDI experiments targeted frozen-hydrated nuclei, we had no information regarding the time-dependent variation of the structural organization of chromosomes. Microscopic imaging study monitored the positions of fluorescent-labeled protein attached to chromosomes demonstrated the high mobility of chromosomes, except the constrained centromeres and telomeres, in the nuclei of budding yeast in the interphase^57^. The mobility of chromosomes may be one of causes for the variation of the fine structures in type-α and type-β maps, such as the tails. In dynamical movement, folding of chromatins into substructural domains and particularly the self-similar arrangement is advantageous to reduce the occurrence of topological entanglement of chromatins, which inhibits gene expression and chromosome segregation.

Regarding the nucleolus, an RNA-rich region, fluorescence imaging studies indicated the location and volume in the nuclei and the condensation of DNA, RNA and proteins. However, we missed any large and C-shaped electron density interpretable as the nucleolus expected from the fluorescence imaging studies^41^ near the periphery of the projection maps (Fig. 3C). One of possible interpretations was that the electron density contrast of the nucleolus (or the concentration of DNA, RNA and proteins) may be lower than that of the substructural domains. According to the schematic model shown in Fig. 1C, the nucleolus may occupy the outside of the CT in the putative models.

Here we compare the structural parameters determined in this study for *S. cerevisiae* with those in previously reported models and structures obtained by other imaging techniques for various biological species of eukaryote including, of course, *S. cerevisiae*. Regarding the substructural domain, the size expected from the *R*s value for a spherical shape (150 nm, Fig. 5A) was close to the dimension of the 160-nm assembly observed in a super-resolution imaging study for human nuclei^12^. In addition, the speculated amount of DNA composing the substructural domain (approximately 450 k bp, Fig. 5A) was consistent with those of TADs found in mammalian nuclei^21–23^. Analyses of chromosomes using Hi-C techniques suggested the fractal property with the power law between the distance and genome size in human nuclei^16^. The fractal nature from the present diffraction study (Fig. 4) did not contradict the fractal globule model, but structural models at higher resolution would be necessary to discuss the power law in detail. The consistency among the results from different imaging techniques for different biological species implies that 150-nm assemblies each containing 450 k bp and the fractal nature of the assembles may be conserved through the evolution of eukaryote.

The nonuniform distributions of electron densities was interpreted as the self-similarly distributed substructural domains in chromosomes (Fig. 5). Here we compared the projection maps with the structural models for chromosome organization in *S*. *cerevisiae* nuclei proposed using 3C and HiC techniques^24,25^. In the 3C model^24^, as 16 chromosomes are hollowly and homogeneously arranged in the vicinity of nuclear membrane, the 3C model has the electron density in the projection map will be high at the periphery and low in the middle. In addition, each chromosome compactly packed into globular shapes without any substructural domains. Therefore, the maps and models from the present X-ray study was inconsistent with the previously proposed 3C model regarding the organization patterns of chromosomes and the presence of the substructural domains. In contrast, in the model from the Hi-C analysis, the centromeres of the chromosomes were closely located near the SPB and extended to nuclear envelop. Therefore, the Hi-C model had similar structural characteristics to the projection maps (Fig. 3C) and models in Fig. 5.

To clarify the inconsistency regarding the structural organization of chromosomes and power law between the distance and genome size, diffraction data is necessary to be accumulated for different views of nearly the same structures to analyze the details of structures using manifold learning^37^ and to visualize common architecture in chromosome organization through the reconstruction of the averaged 3D structures as reported for cyanobacteria cell^39^. The 3D structures of the nuclei at the checkpoints during the cell cycle can be reconstructed based on the cryogenic XDI tomography experiments on frozen-hydrated yeast cells using synchrotron X-rays at a resolution of approximately 25 nm^35^.

## Methods

### Preparation of nuclei

*S. cerevisiae* strain BY4741 (MAT**a** *his3Δ1 leu2Δ0 met15Δ0 ura3Δ0*) was grown at 303 K in a medium containing 1%(w/v) yeast extract (Becton, Dickinson and Company, MA, USA), 2%(w/v) bacterial peptone (Becton, Dickinson and Company, MA, USA), and 2%(w/v) D-glucose (TCI, Tokyo, Japan). When the optical density of the culture medium at 600 nm reached approximately 0.5, cells were harvested and resuspended in another medium containing 1%(w/v) yeast extract, 2%(w/v) peptone, and 2%(w/v) raffinose (TCI, Tokyo, Japan)^58^. After incubation for 16 h at 303 K, the harvested cells were suspended in the same medium and incubated further for 1.5 h at 303 K.

The amount of DNA in the cultivated cells was examined using flow cytometry. Cells from 1 mL culture were fixed with 70% ethanol. After washing with a buffer containing 0.2 M Tris HCl (Wako, Osaka, Japan), 20 mM ethylenediaminetetraacetic acid (Takara, Kusatsu, Japan), and 0.1%(w/v) sodium azide (Wako, Osaka, Japan) (pH 7.5), the cells were incubated for 2 h at 310 K in the presence of 2 mg/mL RNase (Sigma-Aldrich, MA, USA). After exchange with phosphate-buffered saline, DNA was labeled with 4',6-diamidino-2-phenylindole (DAPI) (ImmunoBioScience Corp., WA, USA). The fluorescence from the labeled cells was measured using CytoFLEX (Beckman Coulter, CA, USA). As a result, we observed that 85% of the cells were in the interphase (Fig. 1B).

Nuclei were isolated according to a previously reported protocol^59,60^ with modifications. Before harvesting yeast cells, 0.13% (w/v) sodium azide and 0.5 mM phenylmethylsulfonyl fluoride (PMSF) (Wako, Osaka, Japan) were added to the cell culture. Zymolyase (Nacalai Tesque, Osaka, Japan) at a concentration of 10 mg/mL was used to convert the cells into spheroplasts. After a step-gradient centrifugation of spheroplast lysate, pellets of crude nuclei were suspended in a buffer containing 18%(w/v) Ficoll (Wako, Osaka, Japan), 20 mM PIPES (Dojin Do, Kumamoto, Japan), 5 mM MgCl_2_ (Wako, Osaka, Japan), and 1 mM PMSF (Wako, Osaka, Japan) (pH 6.5), and again centrifuged at 3000 × g for 30 min to remove unlysed cells, cell wall debris, and entrapped membranes. The average diameter of the isolated nuclei was found to be smaller than 1 μm using light microscopy. Although Ficoll was used without monovalent ions in this preparation, little shrinkage of nuclei was observed in reconstructed projection electron density maps (Fig. 3) in comparison with the size observed in TEM (Fig. 1B) and that of the nucleus in *S. cerevisiae* cell using soft-X-ray imaging^30^. Therefore, osmotic deformation by 18%(w/v) Ficoll may be negligible when viewing at the present spatial resolution.

### Specimen preparation for XFEL-XDI experiments

We used a custom-made specimen disk, which was an 8 × 10-mm^2^ silicon frame with nine 1 × 1-mm^2^ windows covered by a silicon–nitride (Si_3_N_4_) membrane with 100 nm thickness (Norcada, Edmonton, Canada)^46^ (Fig. 1C). The silicon–nitride membranes were carbon-coated and further decorated with poly-L-lysine (PLL) (Sigma-Aldrich, MA, USA) to increase the affinity of the membranes to the nuclei.

A 30 μL droplet of suspension of isolated nuclei was placed on the silicon–nitride membranes in a custom-made humidity-controlled chamber^46,61^ mounted on a light microscope X-71 (Olympus, Tokyo, Japan). The relative humidity inside the chamber was maintained at > 90% by supplying moist air from a generator HUM-1 (RIGAKU, Tokyo, Japan). Within a few minutes, the nuclei were adsorbed onto the PLL-decorated silicon nitride membranes. The specimen disk was transferred to a plastic Petri dish, in which the humidity was controlled at 95% with a sponge containing saturated KCl solution.

After adsorption of nuclei onto the membrane for approximately 5 min, the excessive suspension was removed using an MS-B100 spin coater (Mikasa, Tokyo, Japan). The average number density of nuclei remaining on the membranes was approximately 7/10 × 10 μm^2^, as assessed by reference observation of fluorescence from nuclei labeled with DAPI. Finally, each specimen disk was flash-cooled using liquid ethane and stored in liquid nitrogen until further use (Fig. 1C).

TEM images of the nuclei dispersed on carbon membrane were taken using a JEM-2100 electron microscope (JEOL, Tokyo, Japan) operated at an accelerating voltage of 200 kV (Fig. 1B). The TEM image delineates the particle shape only, due to the weak penetration power of electrons. However, the images were usable as references for the shape of the projection electron density maps retrieved from diffraction patterns collected in XFEL-XDI experiments described below.

### XFEL-XDI experiment

Diffraction patterns were collected using our custom-made diffraction apparatus TAKASAGO-6, the MPCCD-Octal and MPCCD-Dual detectors at the beamline 3 of the X-ray free electron laser facility SACLA^42^ (Fig. 1C). The MPCCD-Octal and Dual detectors were tandemly placed 1.6 and 3.2 m downstream the specimen position, respectively. We used focused XFEL pulses which were provided at a repetition rate of 30 Hz and a 10-fs duration. The photon energy of XFEL pulse was 5.5 keV (X-ray wavelength of 0.225 nm). The focused XFEL pulses had almost complete spatial coherence and an ultimately strong intensity at the specimen position (approximately 10^10^ X-ray photons/2 × 2 μm^2^/pulse)^62^.

Specimen disks stored in a liquid nitrogen bath were transferred to the specimen stage inside the vacuum chamber of the diffraction apparatus without frosting and temperature increase^47^. The stage was kept at approximately 80 K during XFEL-XDI experiment. The stage was moved at a maximum speed of 50 μm/33 ms to supply frozen-hydrated nuclei into the focal spot of XFEL pulses. We used the signal from the control system of the SACLA linear accelerator to trigger both the translational motion of the stage and the acquisition of diffraction patterns by the two MPCCD detectors^47^.

Diffraction patterns recorded by the two MPCCD detectors were automatically processed using a custom-made data processing program suite *SITENNO*^48^. After discarding diffraction patterns with respect to the signal-to-noise ratio at a specified resolution, patterns of the two detectors were merged by taking the beam center positions in each detector and the attenuation factor of an aluminum foil placed in front of the MPCCD-Dual detector (Fig. 1C). We used a pattern-matching algorithm to determine the beam center position in each detector as the center of the centrosymmetry in each diffraction pattern.

No diffraction patterns from hexagonal ice particles, which composed of six strong streaks in small-angle region^34^, were observed throughout the two XFEL-XDI experiments, and no images of hexagonal ice particles were reconstructed in the projection maps in the structure analysis. These facts indicated that water in and around nuclei were vitrified by flash-cooling using liquid ethane.

### Processing of diffraction patterns and phase-retrieval calculation

We selected diffraction patterns, which had speckle peaks with a good signal-to-noise ratio beyond a resolution of 33 nm (corresponding to a resolution of 30 μm^‒1^ in diffraction space) and displayed good visibility and centrosymmetry (Fig. 2). The centrosymmetry was evaluated by using the score defined as^48^:

3 $$C_{{{\text{sym}}}} \, = \,\frac{{E^{2} - O^{2} }}{{E^{2} + O^{2} }} \, E\, = \,\frac{1}{2}\sum\limits_{x,y} {\left[ {I\left( {\mathbf{S}} \right) + I\left( { - {\mathbf{S}}} \right)} \right]} {, }O = \frac{1}{2}\sum\limits_{x,y} {\left[ {I\left( {\mathbf{S}} \right) - I\left( { - {\mathbf{S}}} \right)} \right]} ,$$

where $I\left( {\mathbf{S}} \right)$ is the intensity in a targeted region of interest (ROI) and $I\left( { - {\mathbf{S}}} \right)$ is the intensity in the symmetry mate with respect to the beam center position (SI Appendix Fig. S2A). In this study, ROIs for calculating *C*_sym_ were placed in a resolution range of 135 to 64 nm (corresponding to a resolution range of 7.4 to 15.5 μm^−1^ in diffraction space). The maximum resolution of a diffraction pattern was defined as the highest resolution shell, where the signal-to-noise ratios of the speckle peaks were greater than 3. In the two experiments, we obtained 1333 diffraction patterns displayed the maximum resolution beyond 25 nm (corresponding to a resolution range of 40 μm^-1^ in diffraction space), that was near the highest resolution achieved by the specimen-to-detector distance described above.

For each of the selected diffraction patterns, projection electron density maps were reconstructed using two-step phase-retrieval (PR) calculations^63^. In the first step, the hybrid-input–output algorithm^64^ in combination with the shrink-wrap algorithm^65^ was applied to the diffraction pattern trimmed up to a resolution of 51.2 nm (corresponding to a resolution of 19.5 μm^−1^ in diffraction space). In the first step, we determined the most probable support, i.e. particle shape of each nucleus. PR calculations frequently yield non-realistic maps due to the lack of the very small-angle region by the beamstop and Poisson noise in X-ray detection particularly in the high-angle region. Therefore, the most probable support was extracted from 10 groups of retrieved maps separated by K-means clustering after principal component analysis for 700 independently retrieved projection maps^63^. In the second step, 500 electron density maps inside the support selected in the first step were independently retrieved from the diffraction pattern trimmed at a resolution of 25 nm (corresponding to 40 μm^−1^ in diffraction space) using the oversampling smoothness (OSS) algorithm^66^.

Next, we screened the OSS retrieved 500 maps by referring to the similarity score. The similarity score between a pair of maps^51^ is defined as

4 $$T_{{{\text{ij}}}} = \frac{{\sum\limits_{x,y} {\left| {\rho_{{\text{i}}} \left( {x,y} \right) - \rho_{{\text{j}}} \left( {x,y} \right) \, } \right|} }}{{\sum\limits_{x,y} {\left| {\rho_{{\text{i}}} \left( {x,y} \right) + \rho_{{\text{j}}} \left( {x,y} \right) \, } \right|} }},$$

where $\rho_{{\text{i}}} \left( {x,y} \right)$ is the electron density distributions in the *i*-th map. When a pair of maps yields a score of less than 0.2, they are deemed realistic in many cases^34,37–39^.

After PR calculations for 1,333 diffraction patterns, we extracted maps for illustrating the structures of nuclei through the following two steps. First, we examined whether the frequency distribution of $T_{{{\text{ij}}}}$ values for all pairs of the 500 OSS-retrieved maps had a single peak and the center of the distribution was smaller than 0.2 (SI Appendix Fig. S2B). As a typical example shown Fig. S2B, most of the maps displayed sponge-like electron density distribution. For the maps passed the first examination, we examined whether one of the pair of maps that gave the smallest $T_{{{\text{ij}}}}$ had the overall shapes approximated as elliptic circles as observed by TEM (Fig. 1B), and whether the electron density profiles were smooth as shown in Fig. 3B. As a result, approximately 72% of 1333 retrieved maps were discarded, and 373 maps passed the two-step examination. The frequency distribution for the smallest $T_{{{\text{ij}}}}$ values for the selected maps is shown in SI Appendix Fig. S2C.

A set of structure amplitudes calculated from the realistic maps ($\left| {F_{{{\text{obs}}}} \left( {\mathbf{S}} \right)} \right|$) were compared with those experimentally observed ($\left| {F_{{{\text{calc}}}} \left( {\mathbf{S}} \right)} \right|$) using the crystallographic *R*-factor defined as

5 $$R_{{\text{F}}} = \frac{{\sum\limits_{{\mathbf{S}}} {\left| {\left| {F_{{{\text{obs}}}} \left( {\mathbf{S}} \right)} \right| - K\left| {F_{{{\text{calc}}}} \left( {\mathbf{S}} \right)} \right|} \right|} }}{{\sum\limits_{{\mathbf{S}}} {\left| {F_{{{\text{obs}}}} \left( {\mathbf{S}} \right)} \right|} }},$$

where $K$ is a scale factor. The average crystallographic *R-*factor of the selected maps was 0.18 (SI Appendix Fig. S2D).

## Supplementary Information

Supplementary Information.

## Data Availability

The datasets used and/or analyzed during the current study available from the corresponding author on reasonable request.
