# Supplementary material for: Ultrastructure and fractal property of chromosomes in close-to-native yeast nuclei visualized using X-ray laser diffraction
Source: Sci Rep. 2023 Jul 5;13:10802. doi: 10.1038/s41598-023-37733-6 (PMC10322978; doi:10.1038/s41598-023-37733-6)
Supplement: Supplementary file 1 — Supplementary Information. [file 41598_2023_37733_MOESM1_ESM.docx]

Supporting Information for

Ultrastructure and fractal property of chromosomes in close-to-native yeast nuclei visualized using X-ray laser diffraction

So Uezu^1,2^, Takahiro Yamamoto^1,2^, Mao Oide^1,2,3^, Yuki Takayama^2,4,5,6^, Koji Okajima^1,2^, Amane Kobayashi^1,2^, Masaki Yamamoto^2^, Masayoshi Nakasako^1,2,*^

^1^Department of Physics, Faculty of Science and Engineering, Keio University, 3-14-1 Hiyoshi, Kohoku-ku, Yokohama, Kanagawa 223-8522, Japan.

^2^RIKEN Spring-8 Center, 1-1-1 Kouto, Sayo-cho, Sayogun, Hyogo 679-5148, Japan.

^3^PRESTO, Japan Science and Technology Agency, Chiyoda-ku, Tokyo, 102-0076, Japan

^4^Graduate School of Science, University of Hyogo, 3-2-1 Kouto, Kamigori-cho, Ako-gun, Hyogo 678-1297, Japan.

^5^International Center for Synchrotron Radiation Innovation Smart, Tohoku University, Katahira 2-1-1, Aoba-ku, Sendai 980-8577, Japan.

^6^CRESTO, Japan Science and Technology Agency, Chiyoda-ku, Tokyo, 102-0076, Japan

*To whom correspondence should be addressed.

Phone: +81-45-566-1713. Fax: +81-45-566-1672.

E-mail: nakasako@phys.keio.ac.jp.

**Table S1. Composition of chromosomes in budding yeast**

| chromosome | size (bp) | chromosome | size (bp) |
| --- | --- | --- | --- |
| I | 230,218 | IX | 439,888 |
| II | 813,184 | X | 745,751 |
| III | 316,620 | XI | 666,816 |
| IV | 1,531,933 | XII | 1,078,177 |
| V | 576,874 | XIII | 924,431 |
| VI | 270,161 | XIV | 784,333 |
| VII | 1,090,940 | XV | 1,091,291 |
| VIII | 562,643 | XVI | 948,066 |

**Reference**

Goffeau, A., Barrell, B. G., Bussey, H., Davis, R. W., Dujon, B., Feldmann, H., Galibert, F., Hoheisel, J. D., Jacq, C., Johnston, M., Louis, E. J., Mewes, H. W., Murakami, Y., Philippsen, P., Tettelin, H. & Oliver, S. G. *Science* **1996**, *274*, 546, 563-567 (1996).

**
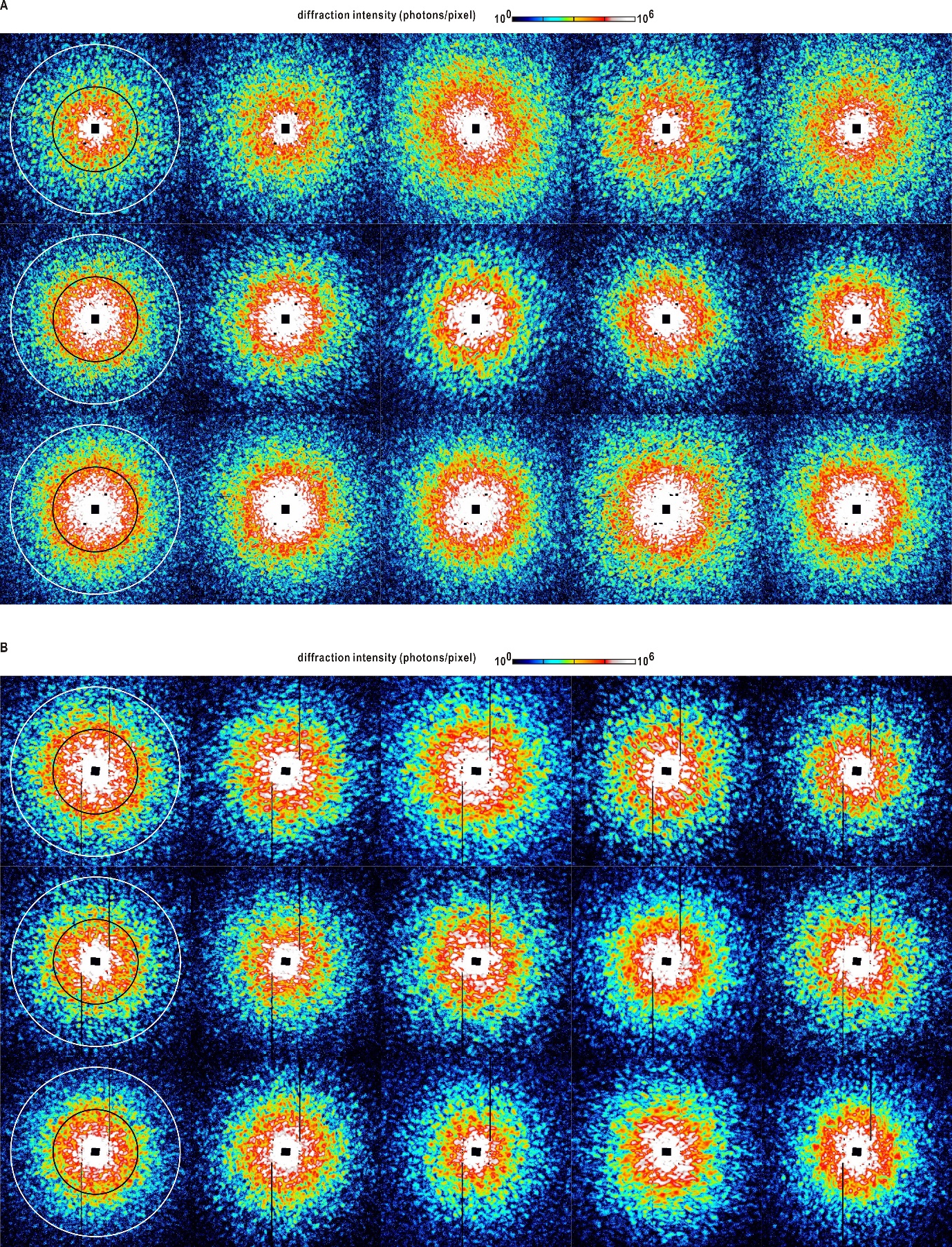
**

**Figure S1**

Diffraction patterns from the yeast nuclei in the interphase collected in the first (**A**) and second (**B**) experiments. The black and white circles in the left column indicate resolution limits of 50 and 33 nm (corresponding to 20 and 30 μm^-1^ in diffraction space), respectively.


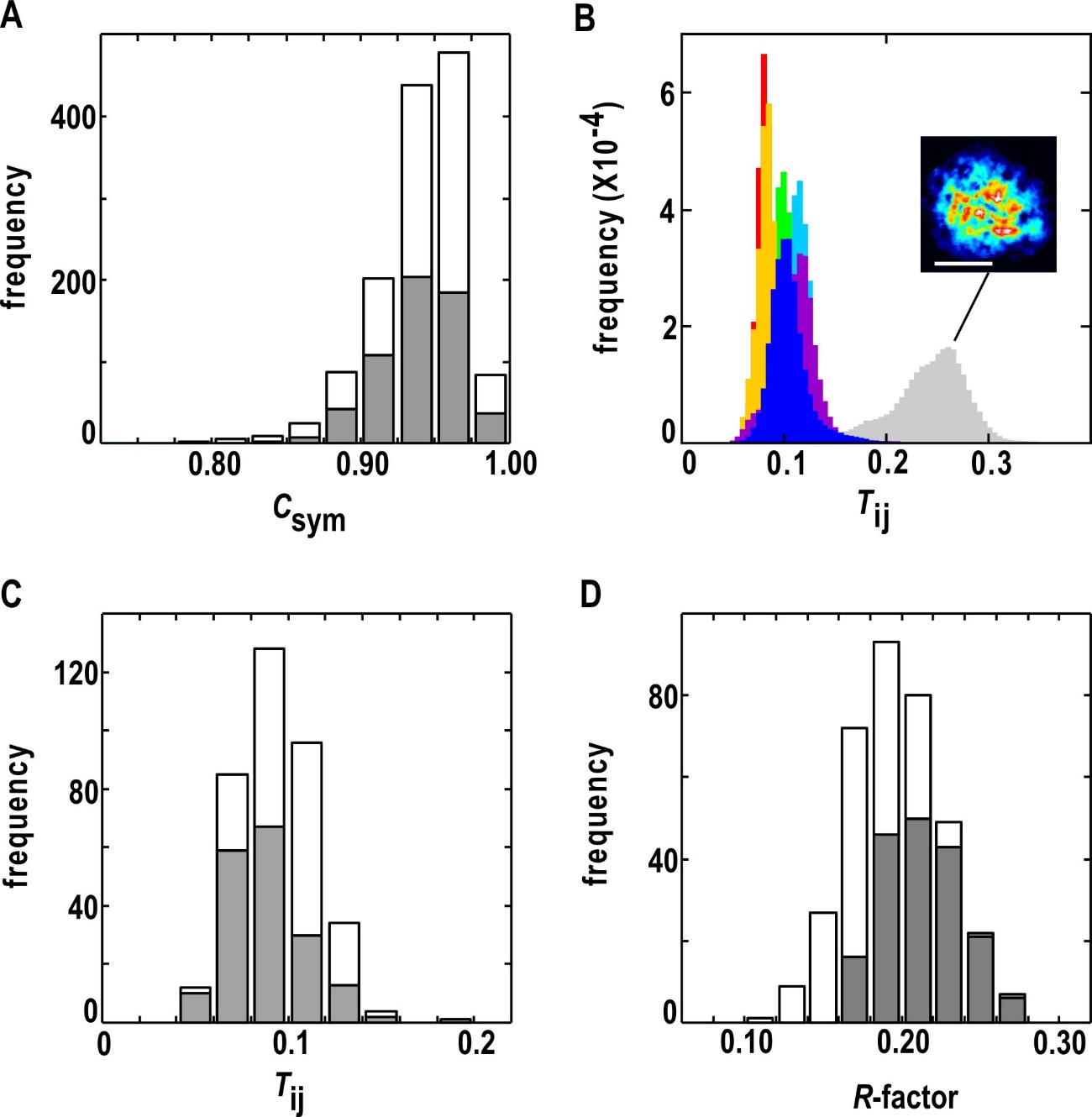


**Figure S2**

(**A**) Frequency distribution on the centrosymmetry scores, *C*_sym_, for 1,333 high quality diffraction patterns. In all panels, the gray and white bars indicate data from the first and second experiments, respectively.　(**B**) Frequency distributions of the similarity scores (*T*_ij_) for all pairs of 500 maps independently retrieved from each diffraction pattern. In the case of successfully retrieved six maps (red, orange, green blue, cyan, purple and gray bars), the *T*_ij_ values were smaller than 0.2, and the peaks of the frequency distributions were located around 0.1. The maps with the smallest *T*_ij_ values had elliptic shapes and the internal structures shown in Fig. 3C in the main text. In contrast, regarding one failure case (gray bars), the most of the *T*_ij_ values were larger than 0.2, and the peak of the frequency distribution was located around 0.25. Even the map with the smallest score had a sponge-like structure. The scale bar is 500 nm. The clear differences of the distribution between the correctly and incorrectly retrieved maps demonstrates the effectiveness of the score to screening maps with realistic shapes and internal structures. Frequency distributions of the *T*_ij_ values (**C**) and crystallographic *R*-factors (**D**) of the selected 373 maps.

**
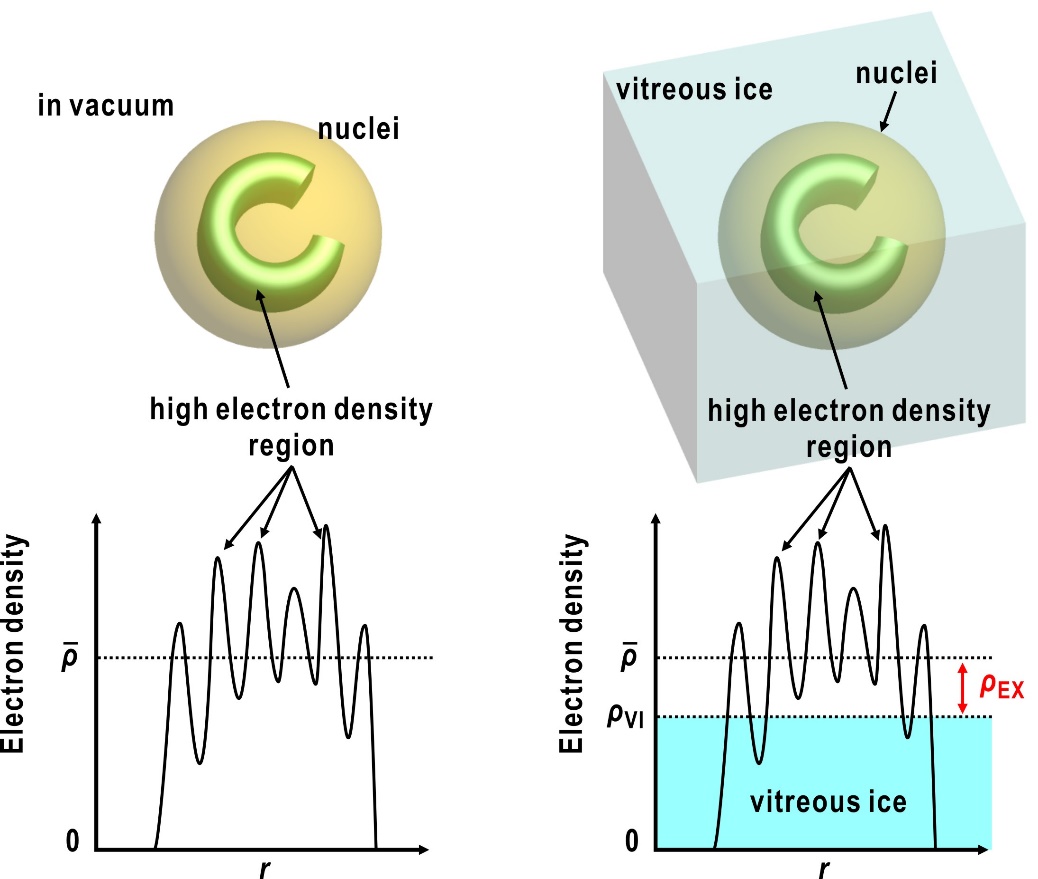
**

**Figure S3**

**Electron density contrast between nuclei and vitreous ice**

In our X-ray diffraction imaging (XDI) experiments, the specimen particles were embedded in vitreous ice. In the resolution range of 400-20 nm, the projected electron density distribution of vitreous ice can be approximated as a constant ($\rho_{\text{sol}}$). Then, the projected electron distribution $\rho\left(\vec{r}\right)$ (Fig. S4) can be expressed using $\rho_{\text{sol}}$ and electron density $\rho_{\text{ex}}\left(\vec{r}\right)$ of a particle excess from $\rho_{\text{sol}}$ as

$\rho\left(\vec{r}\right)=\rho_{\text{sol}}+\rho_{\text{ex}}\left(\vec{r}\right),\text{ }\rho_{\text{ex}}\left(\vec{r}\right)=\Delta\rho\text{ }\rho_{\text{C}}\left(\vec{r}\right)+\rho_{\text{F}}\left(\vec{r}\right),\text{ }\Delta\rho=\bar{\rho}-\rho_{\text{sol}}$, (S7.1)

where $\rho_{\text{C}}\left(\vec{r}\right)()$ expresses the shape of the particle with the average electron density $\bar{\rho}$, and takes the value of 1 inside and 0 outside the particle. $\rho_{\text{F}}\left(\vec{r}\right)()$ is the spatial fluctuation of electron density from the average $\bar{\rho}$ (Ibel & Stuhrmann, 1975).

Then, the diffraction intensity from the particle and vitreous ice within the incident X-ray beam size of incident X-rays at a scattering vector $\vec{S}$, $I\left(\vec{S}\right)()$, can be expressed as

$I\text{ }\left(\vec{S}\right)\propto4\pi^{2}\rho_{\text{sol}}^{2}\delta\left(\vec{S}\right)^{2}+\Delta\rho^{2}\text{ }\left|\text{ }F_{\text{C}}\left(\vec{S}\right)\text{ }\right|^{2}+\Delta\rho\text{ }F_{\text{C}}^{*}\left(\vec{S}\right)\text{ }F_{\text{F}}\left(\vec{S}\right)+\Delta\rho\text{ }F_{\text{F}}^{*}\left(\vec{S}\right)\text{ }F_{\text{C}}\left(\vec{S}\right)+\left|\text{ }F_{\text{F}}\left(\vec{S}\right)\text{ }\right|^{2},$ (S7.2)

where $\delta\left(\vec{S}\right)$ is Dirac delta function. $F_{\text{C}}\left(\vec{S}\right)()$ and $F_{\text{F}}\left(\vec{S}\right)()$ are the structure factors of $\rho_{\text{C}}\left(\vec{r}\right)()$ and $\rho_{\text{F}}\left(\vec{r}\right)()$, respectively. As the first term is zero except for $\vec{S}=0$, X-rays diffracted by the vitreous ice outside the particle go into a beam stop. Then, the electron density outside the particle becomes small in the projection map retrieved from the diffraction pattern. In addition, the diffraction intensity is dominated by $\left|\text{ }F_{\text{F}}\left(\vec{S}\right)\text{ }\right|^{2}$when $\Delta\rho$ becomes small. For instance, $\bar{\rho}$ values of nucleic acids, proteins, and water (vitreous ice) are 0.55, 0.42, and 0.33 electrons Å^-3^, respectively (Stuhrmann & Miller, 1978). Therefore, the projection maps of cells in vitreous ice are dominated by the internal structure, $\rho_{\text{F}}\left(\vec{r}\right)()$, with a high electron density region rather than the overall shapes in the projection maps. In contrast, for dried specimens in a vacuum, the shape term is dominant, as reported previously (Kobayashi *et al*., 2016a).

The influence of electron density contrast has been examined in our previous simulation studies on XDI for macromolecules in vitreous ice or water (Kodama & Nakasako, 2011; Oroguchi & Nakasako, 2013). As demonstrated in previous studies, the border of macromolecules is blurred by the electron density contrast between the macromolecules and vitreous ice. In addition, the same effect was confirmed in the XDI structure analysis of cellular organelles and cells embedded in vitreous ice (Nakasako *et al*., 2013; Oroguchi *et al*., 2015; Takayama *et al*., 2015; Kobayashi *et al*. 2016a; Kobayashi *et al*. 2016b; Kobayashi *et al*., 2018; Kobayashi *et al*., 2021).

**References**

Ibel, K. & Stuhrmann, H.B. *J. Mol. Biol*. **93**, 255-265 (1975).

Stuhrmann, H.B. & Miller, A. *J. Appl. Crystallogr.* **11**, 325–345 (1978).

Kodama, W. & Nakasako, M. *Phys. Rev*. E 84, 021902 (2011).

Oroguchi, T. & Nakasako, M. *Phys. Rev*. E 87, 022712 (2013).

Nakasako, M., Takayama, Y., Oroguchi, T., Sekiguchi, Y., Kobayashi, A., Shirahama,K., Yamamoto, M., Hikima, T., Yonekura, K., Maki-Yonekura, S., Kohmura, Y., Inubushi, Y., Takahashi, Y., Suzuki, A., Matsunaga, S., Inui, Y., Tono, K., Kameshima, T., Joti, Y., & Hoshi, T. *Revi. Sci. Instrum.* **84**, 093705 (2013).

Oroguchi, T., Sekiguchi, Y., Kobayashi, A., Masaki, Y., Fukuda, A., Hashimoto, S., Nakasako, M., Ichikawa, Y., Kurumizaka, H., Shimizu, M., Inui, Y., Matsunaga, S., Kato, T., Namba, K., Yamaguchi, K., Kuwata, K., Kameda, H., Fukui, N., Kawata, Y., Kameshima, T., Takayama, Y., Yonekura, K. & Yamamoto, M. *J. Phys.* B **48**, 184003 (2015).

Takayama, Y., Inui, Y., Sekiguchi, Y., Kobayashi, A., Oroguchi, T., Yamamoto, M., Matsunaga, S. & Nakasako, M. *Plant Cell Physiol.* **56**, 1272-1286 (2015).

Kobayashi, A., Sekiguchi, Y., Oroguchi, T., Okajima, K., Fukuda, A., Oide, M., Yamamoto, M. & Nakasako, M. *J. Synchrotron Rad.* **23**, 975–989 (2016a).

Kobayashi, A., Sekiguchi, Y., Takayama, Y., Oroguchi, T., Shirahama, K., Torizuka, Y., Manoda, M., Nakasako, M. & Yamamoto, M. *Rev. Sci. Instrum.* **87**, 053109 (2016b).

Kobayashi, A., Takayama, Y., Okajima, K., Oide, M., Yamamoto, T., Sekiguchi, Y., Oroguchi, T., Nakasako, M., Kohmura, Y., Yamamoto, M., Hoshi, T. & Torizuka, Y. *J. Synchrotron Rad.* **25**, 1803–1818 (2018).

Kobayashi, A., Takayama, Y., Hirakawa, T., Okajima, K., Oide, M., Oroguchi, T., Inui, Y., Yamamoto, M., Matsunaga S. & Nakasako, M. *Sci. Rep.* **11**, 3877 (2021).
